# A Survey of the Holistic Healthcare Approach by Healthcare Providers at a Tertiary Care Teaching Hospital

**DOI:** 10.7759/cureus.105633

**Published:** 2026-03-22

**Authors:** Ruchita J Mer, Gaurav G Kakasaniya, Tejas A Acharya, Madhav D Trivedi, Sunita B Chhaiya, Dimple S Mehta

**Affiliations:** 1 Pharmacology, C. U. Shah Medical College and Shrimad Rajchandra Sarvamangal Hospital, Surendranagar, IND

**Keywords:** healthcare provider, holistic healthcare, holistic medicine, holistic patient care, holistic patient management

## Abstract

Background

A holistic approach to healthcare treats the full person, not just the sickness or symptoms. This means taking care of their physical, emotional, social, cultural, and spiritual needs. The relationship between healthcare providers and patients is built on respect, openness, equality, and mutuality. We conducted this study to assess the knowledge, attitudes, and practices about the holistic healthcare approach among healthcare workers at a tertiary care teaching hospital.

Methodology

A prospective, questionnaire-based survey study was conducted at a tertiary care teaching hospital after Institutional Ethics Committee approval. Data were collected over two months (October-November 2025) using a validated questionnaire covering three domains (knowledge, attitude, and practice), which was distributed through Google Forms (Google LLC, Mountain View, CA, USA). Data were analyzed using descriptive statistics such as frequencies and percentages, and inferential statistics were performed using the chi-square test.

Results

A total of 222 healthcare providers participated. Doctors comprised 128 (57.7%), and nursing staff comprised 94 (42.3%) participants. Overall, 222 (100%) respondents correctly answered that all healthcare team members (doctors, nurses, physiotherapists, etc.) should provide holistic care. The majority expressed agreement that emotional support is as important as medicines, with 101 (45.5%) strongly agreeing. In practice, most participants addressed emotional concerns and involved family members; however, 183 (82.4%) had not received formal training in holistic healthcare. The chi-square test showed that the answers to the statements about attitude and questions about practice were statistically different (p < 0.05).

Conclusions

Healthcare providers have excellent knowledge and positive attitudes toward holistic healthcare, with generally good practices. However, limited formal training and workload barriers highlight the need for regular training programs to strengthen holistic care delivery.

## Introduction

The pursuit of comprehensive well-being and the preservation of good health have been perennial objectives of human civilization. A significant transition toward a holistic strategy that incorporates both necessary nutrients and pharmaceuticals has surfaced as a potential paradigm in healthcare. This all-encompassing view recognizes that health is more than just not having a sickness. It stresses the need to look at the whole person, including their physical, mental, and emotional health [1].

Holistic healthcare shifts the focus from isolated diagnosis and treatment to a comprehensive approach that encompasses the entire cycle of care [2]. In holistic medicine, the focus is not only on treating illness but also on promoting health and preventing illness, with a strong emphasis on education. Holistic practitioners see health as a range that goes from being clinically ill to being completely healthy in body, mind, and spirit. They work toward the idea of “super health,” which is when someone has amazing energy, happiness, and creativity. This method helps people reach their full biopsychosocial potential and deal with their medical problems. Holistic nursing takes into account all aspects of a patient’s life, including how their ideas, feelings, cultures, beliefs, and attitudes affect their recovery, happiness, and fulfillment. Respecting human dignity is the basis of comprehensive care. It also means building relationships based on equality, respect, openness, and shared decision-making [3].

Respect, openness, equality, and mutuality are the foundations of the relationship between healthcare practitioners and patients. Healthcare professionals view a patient holistically, acknowledging the integration of body, mind, and spirit within their surroundings. Another part of holistic care is respecting the patient’s position in the treatment process, having them participate in it, and encouraging self-care. This leads to therapeutic consultation, hope, dignity, self-discipline, social progress, a sense of autonomy, vigor, and vitality [4].

Healthcare professionals play an important role in the successful implementation of holistic healthcare. Their knowledge, attitudes, and practices significantly influence patient experiences, treatment adherence, and overall health outcomes. A positive attitude toward holistic care and adequate knowledge of its principles are essential for integrating holistic approaches into routine clinical practice.

However, despite growing interest in holistic healthcare, there is limited evidence regarding the knowledge, attitudes, and practices of healthcare professionals toward holistic care, particularly in tertiary care teaching hospitals in developing healthcare settings. Therefore, the present study aimed to assess the knowledge, attitudes, and practices about the holistic healthcare approach among healthcare professionals at a tertiary care teaching hospital.

## Materials and methods

Study design and site

A questionnaire-based, prospective survey was conducted after taking approval from the Institutional Ethics Committee (approval number: CUSMC/IEC(HR)/RP/27/2025/Final Approval/239/2025) at C. U. Shah Medical College and Shrimad Rajchandra Sarvamangal Hospital.

Study population, sample size, and study period

The total population of healthcare professionals at C. U. Shah Medical College and Shrimad Rajchandra Sarvamangal Hospital included 226 doctors, 175 resident doctors, and 266 nurses. Thus, the total population of healthcare providers was 667. The sample size was determined using the Lynch formula [5], which resulted in a required sample size of 224 participants. ​Lynch formula: n = (NZ²pq)/(NE²+Z²pq), where n is the required sample size, N is the population size, Z is the Z-value (standard score corresponding to the desired confidence level, e.g., 1.96 for 95%), p is the estimated proportion of the population with the characteristic of interest, q is 1 − p (proportion of the population without the characteristic), and E is the margin of error (acceptable error level).

To ensure proportional representation of all professional categories, stratified random sampling was employed. The study population was first divided into three strata based on professional designation, namely, doctors, resident doctors, and nurses. Within each stratum, randomization was performed. Participants were then selected using the even-odd number method, wherein individuals assigned even serial numbers were included in the study, while those with odd serial numbers were excluded. This method ensured that selection was random and that each eligible participant had an equal probability of being included. The study was conducted over a period of two months (October to November 2025).

Inclusion criteria

The study included doctors, resident doctors, and nurses who voluntarily agreed to participate and provided informed consent for involvement in the study.

Data collection

Healthcare professionals were selected through a stratified random sampling technique and divided into three strata; then, from each stratum, participants with even serial numbers were included. These participants were formally informed about the study through an official WhatsApp Messenger (Meta Platforms, Inc., Menlo Park, CA, USA) containing a direct link to the validated questionnaire. The questionnaire was distributed electronically via Google Forms (Google LLC, Mountain View, CA, USA) to ensure ease of access, participant convenience, and data security.

Data collection was conducted over a continuous two-month period. During this phase, periodic reminder notifications and follow-up messages were systematically disseminated via WhatsApp Messenger to enhance participation and encourage timely responses, thereby improving the response rate.

We specifically developed the questionnaire used for this study for research purposes. It was developed following a review of pertinent literature on holistic healthcare. The questionnaire had items evaluating the knowledge, attitudes, and practices of medical professionals concerning holistic healthcare [6]. The questionnaire consisted of three sections comprising a total of 15 questions. The first section included five questions assessing knowledge related to holistic healthcare. The second section focused on the attitudes of healthcare providers toward holistic healthcare and contained five questions. The third and final section assessed the practices of healthcare providers and included five questions. The prepared questionnaire was further validated by four external faculty members. The Item Content Validity Index (I-CVI) and the Scale Content Validity Index (S-CVI) were used to check the content validity at both the item and scale levels [7]. The faculty members were asked to use a four-point scale to score each item for how relevant, clear, simple, and ambiguous it was. The four-point Content Validity Index (CVI) was used to measure content validity. The S-CVI/Ave approach was used to determine the questionnaire’s overall content validity. The I-CVI values were between 0.75 and 1.00, while the S-CVI/Ave values were between 0.98 and 1.00, indicating a high level of content validity. The finalized questionnaire is presented in Table 1.

**Table 1:** Validated questionnaire for the evaluation of the holistic healthcare approach.

Knowledge
1. What does “holistic care” mean in patient treatment? a) Giving only medicines; b) Treating only physical illness; c) Caring for body, mind, emotions, and social needs of the patient; d) Focusing only on the patient’s religious practices
2. Who should provide holistic care to the patient? a) Only the doctor; b) Only the nurse; c) Only the family; d) All healthcare team members (doctors, nurses, physiotherapists, etc.)
3 Which one is a part of holistic care? a) Only giving injections and medicines; b) Listening to the patient’s problems and emotions; c) Giving bed baths and dressing only; d) Discharge planning only
4. Holistic care is best supported by which of the following strategies? a) Rigid protocol-based care; b) Time-limited patient interaction; c) Active patient listening and individualized care plans; d) Focusing only on lab reports
5. Which one is an example of holistic care? a) Giving only the prescribed tablets on time; b) Talking to patient and family, explaining care, and considering patient’s feelings; c) Checking only blood pressure and pulse; d) Focusing on test results only
Attitude (a) Strongly Agree, b) Agree, c) Neutral, d) Disagree, e) Strongly Disagree)
1. I believe that emotional support is as important as giving medicines
2. I feel confident talking to patients about their personal or emotional problems
3. Holistic care helps patients recover faster and feel better
4. I feel I need more training to give complete holistic care to patients
5. Due to work pressure and a shortage of time, it is difficult to give full holistic care
Practice (a) Always, b) Often, c) Sometimes, d) Rarely, e) Never)
1. How often do you ask patients about their worries or emotional problems?
2. Do you involve the patient’s family while explaining the treatment or care?
3. Do you consider the patient's language, culture, or beliefs while caring for them?
4. When a patient is scared or anxious, do you try to calm or support them?
5. Have you attended any training or workshop on patient communication or holistic care? a) Yes; b) No; c) Not sure

The questionnaire was converted into Google Forms. The Google Forms was subdivided into six sections. The first section comprised the title of the study, along with a brief introduction about the study, and contained the informed consent statement. Participants were required to give consent to proceed to the subsequent sections. The second section comprised demographic details of the participants. This included the initials of the participant’s name, age, gender, profession, and years of experience. The third section comprised knowledge-related questions, containing five multiple-choice questions. These questions were designed to evaluate participants’ understanding and conceptual awareness. The fourth section contained five attitude-related statements measured on a five-point Likert scale ranging from “strongly agree” to “strongly disagree.” The fifth section assessed participants’ practices related to holistic healthcare. It included five multiple-choice questions that evaluated how often participants used holistic healthcare in their clinical practice. Lastly, the sixth section contained a thank-you note to the participants.

Statistical analysis

The data collected were entered into a Microsoft Excel (Microsoft Corp., Redmond, WA, USA) spreadsheet. We used percentages and frequency for descriptive analysis and the chi-square test for inferential statistics. Frequencies and percentages were used to summarize categorical characteristics, including gender, age, years of experience, and profession. The association between knowledge and participants’ gender and profession was evaluated using the chi-square test. For Likert-scale responses related to the attitudes of healthcare providers toward holistic healthcare, data were summarized as frequencies and percentages. To further assess the association between healthcare providers’ attitudes toward holistic healthcare, the chi-square test was applied. Similarly, the association between profession and attitudes toward holistic healthcare was further evaluated using the chi-square test. For responses related to the practices of healthcare providers toward holistic healthcare, data were summarized as frequencies and percentages. To further assess the association between healthcare providers’ practices toward holistic healthcare, the chi-square test was applied. Similarly, the association between profession and practices toward holistic healthcare was further evaluated using the chi-square test. For all inferential statistical analyses, a p-value of less than 0.05 was considered statistically significant. All analyses were conducted using Microsoft Excel, whereas confirmatory tests were performed using Epi Info™ version 7.2 software (Centers for Disease Control and Prevention, Atlanta, GA, USA).

## Results

Demographic details

A total of 224 participants responded to the questionnaire. However, two participants did not provide informed consent and were therefore excluded from the analysis. Consequently, the final sample size consisted of 222 participants. Of these, 129 (58.1%) were male, and 93 (41.9%) were female. Most participants were aged 20-29 years (113, 50.9%). Among the participants, the majority had 1-5 years of experience (110, 49.5%). Most participants were doctors at 128 (57.7%), while nurses accounted for 94 (42.3%) of the study participants, as depicted in Table 2.

**Table 2:** Demographic details of the respondents.

Category	Subcategory	Number (n = 222)
Gender	Male	129 (58.1%)
	Female	93 (41.9%)
Age group (years)	20–29	113 (50.9)
	30–39	77 (34.7%)
	40–49	16 (7.2%)
	50–59	13 (5.9%)
	≥60	3 (1.4%)
Years of experience	<1 year	13 (5.9%)
	1–5 years	110 (49.5%)
	6–10 years	60 (27%)
	11–20 years	23 (10.4%)
	>20 years	16 (7.25%)
Profession	Doctors	128 (57.7%)
	Nurses	94 (42.3)

Knowledge about holistic healthcare

**Figure 1:**
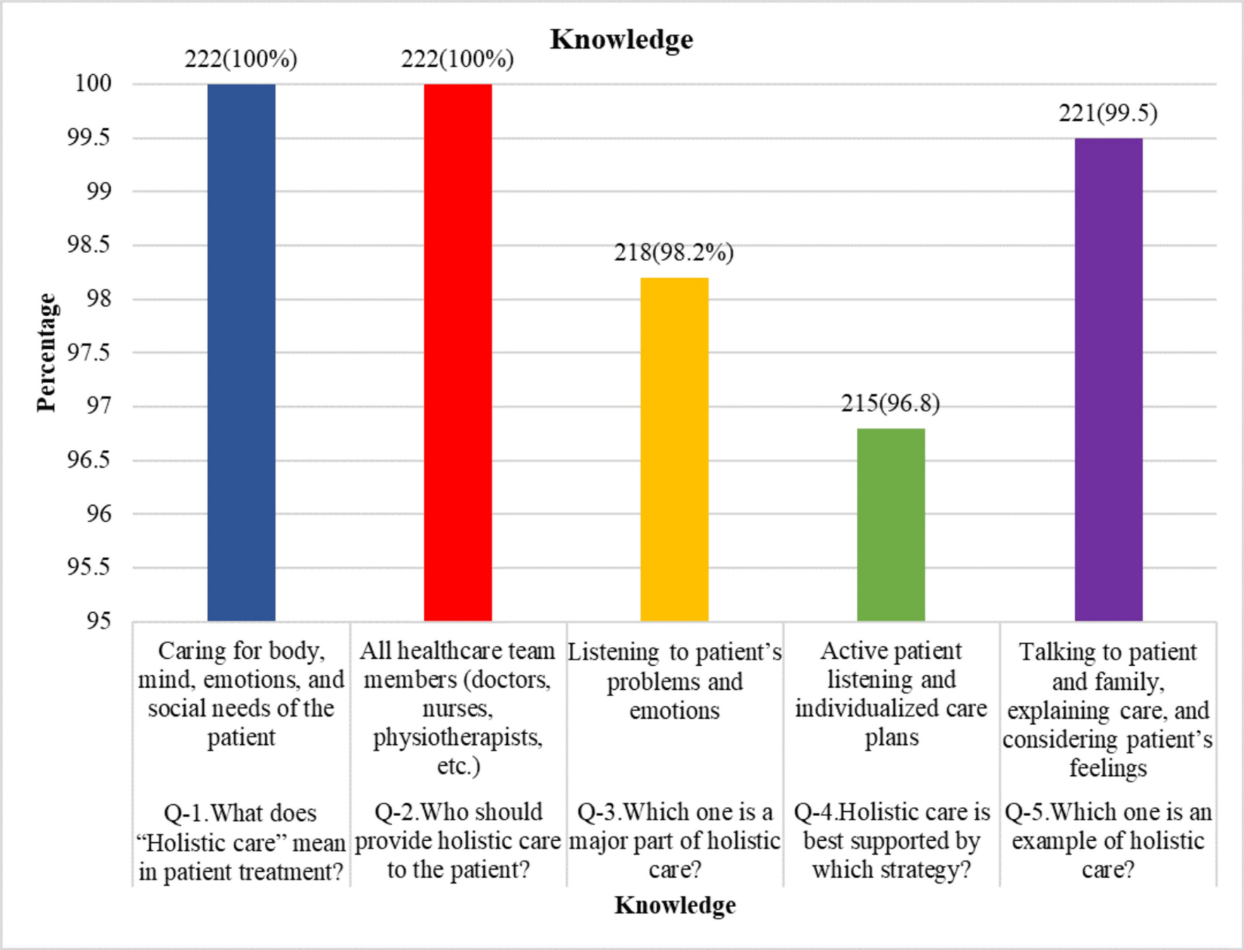
Knowledge about holistic healthcare.

As shown in Figure 1, healthcare providers had a great level of knowledge of holistic care. Overall, 222 (100%) correctly identified the meaning of holistic care as caring for the body, mind, emotions, and social needs of the patient. Further, 222 (100%) respondents correctly answered that all healthcare team members (doctors, nurses, physiotherapists, etc.) should provide holistic care. Additionally, 218 (98.2%) correctly identified listening to patients’ problems and emotions as a major component of holistic care, 215 (96.8%) selected active patient listening and individualized care plans as the best supporting strategy, and 221 (99.5%) recognized talking to patients and families, explaining care, and considering patients’ feelings as an example of holistic care. Mean knowledge of healthcare providers was 98.9%. The chi-square test showed no statistically significant relationship between gender and knowledge level (χ² = 0.17, p = 0.68) when considering a significance level of p < 0.05. Profession and knowledge of holistic care did not significantly correlate (χ² = 2.74, p = 0.098) (Tables 3, 4).

**Table 3:** Gender-wise knowledge about holistic healthcare. Data mentioned in the table represent the number of respondents (n) and the percentage (%). *: χ² value represents the chi-square test statistic; **: p-value <0.05 is considered statistically significant. Participants achieving scores of ≥95% were classified as possessing excellent knowledge, while those scoring between 75% and 94% were classified as having good knowledge.

Gender	Excellent	Good	Total	Statistical test	P-value**
Male (n = 129)	126 (97.7%)	3 (2.3%)	129	χ²* = 0.17	0.68
Female (n = 93)	90 (96.8%)	3 (3.2%)	93		
Total (n = 222)	216 (97.3%)	6 (2.7%)	222		

**Table 4:** Profession-wise knowledge about holistic healthcare. Data mentioned in the table represent the number of respondents (n) and the percentage (%). *: χ² value represents the chi-square test statistic; **: p-value <0.05 is considered statistically significant. Participants achieving scores of ≥95% were classified as possessing excellent knowledge, while those scoring between 75% and 94% were classified as having good knowledge.

Profession	Excellent	Good	Total (n)	Statistical test	P-value**
Nurses	92 (97.9%)	2 (2.1%)	94	χ²* = 2.74	0.098
Doctors	128 (100%)	0 (0%)	128		
Total	220 (99.1%)	2 (0.9%)	222		

Attitude toward holistic healthcare

The findings of our study showed that the majority expressed agreement that emotional support is as important as medicine, with 101 (45.5%) strongly agreeing. Overall, 117 (52.7%) participants agreed that they felt confident about discussing patients’ personal and emotional problems. Most participants acknowledged the benefits of holistic care, with 114 (51.4%) agreeing that it helps with faster recovery and well-being. Further, 121 (54.5%) agreed on the need for more training to provide complete holistic care. Moreover, 108 (48.6%) agreed that work pressure and time shortage make holistic care difficult. Overall, the findings indicate a predominantly positive attitude toward holistic care. The responses to all five attitude statements were statistically significant (χ² test, p < 0.05), indicating that participants predominantly agreed with statements supporting the importance of holistic care (Table 5). For Q2 (χ² = 15.79, p < 0.05), Q4 (χ² = 19.92, p < 0.05), and Q5 (χ² = 26.48, p < 0.05), a chi-square test of independence revealed significant differences between doctors and nurses. However, Q1 (χ² = 0.17, p < 0.05) and Q3 (χ² = 3.57, p < 0.05) did not demonstrate any significant correlation (Table 6).

**Table 5:** Attitude toward holistic healthcare. Data mentioned in the table represent the number of healthcare providers (n) and the percentage (%). *: χ² value represents the chi-square test statistic; **: p-value <0.05 is considered statistically significant.

Attitude statement	Strongly agree	Agree	Neutral	Disagree	Statistical test	P-value**
Q1. I believe that emotional support is as important as giving medicines	101 (45.5%)	83 (37.4%)	38 (17.1%)	0 (0%)	χ²* = 28.46	<0.001
Q2. I feel confident talking to patients about their health-related, personal, and emotional problems	68 (30.6%)	117 (52.7%)	37 (16.7%)	0 (0%)	χ²* = 45.49	<0.001
Q3. Holistic care helps patients recover faster and feel better	62 (27.9%)	114 (51.4%)	45 (20.3%)	1 (0.5%)	χ²* = 97.21	<0.001
Q4. I feel I need more training to give complete holistic care to patients	39 (17.6%)	121 (54.5%)	61 (27.5%)	1 (0.5%)	χ²* = 136.27	<0.001
Q5. Due to work pressure and a shortage of time, it is difficult to give full holistic care	30 (13.5%)	108 (48.6%)	72 (32.4%)	12 (5.4%)	χ²* = 68.76	<0.001

**Table 6:** Profession-wise attitudes toward holistic healthcare. Data mentioned in the table represent the number of doctors and nurses (n) and the percentage (%). *: χ² value represents the chi-square test statistic; **: p-value <0.05 is considered statistically significant.

Question	Profession	Strongly agree	Agree	Neutral	Disagree	Statistical test	P-value**
Q1. Emotional support is as important as medicines	Doctor (n = 128)	57 (44.5%)	48 (37.5%)	23 (18.0%)	0 (0%)	χ²* = 0.17	0.918
	Nurse (n = 94)	44 (46.8%)	35 (37.2%)	15 (16.0%)	0 (0%)		
Q2. Confidence in discussing emotional problems	Doctor (n = 128)	52 (40.6%)	54 (42.2%)	22 (17.2%)	0 (0%)	χ²* = 15.79	<0.001
	Nurse (n = 94)	16 (17.0%)	63 (67.0%)	15 (16.0%)	0 (0%)		
Q3. Holistic care helps patients recover faster	Doctor (n = 128)	42 (32.8%)	59 (46.1%)	26 (20.3%)	1 (0.8%)	χ²* = 3.57	0.312
	Nurse (n = 94)	20 (21.3%)	55 (58.5%)	19 (20.2%)	0 (0%)		
Q4. Need more training for holistic care	Doctor (n = 128)	10 (7.8%)	64 (50.0%)	35 (27.3%)	0 (0%)	χ²* = 19.92	<0.001
	Nurse (n = 94)	29 (30.9%)	57 (60.6%)	26 (27.7%)	1 (1.1%)		
Q5. Work pressure makes holistic care difficult	Doctor (n = 128)	25 (19.5%)	64 (50.0%)	38 (29.7%)	1 (0.8%)	χ²* = 26.48	<0.001
	Nurse (n = 94)	5 (5.3%)	44 (46.8%)	34 (36.2%)	11 (11.7%)		

Practice of holistic healthcare

In our study, most participants reported positive patient-centered practices. The majority always asked patients about emotional concerns (140, 63.1%), involved family members while explaining care (152, 68.5%), and considered patients’ language, culture, or beliefs, with 92 (41.4%) always and 88 (39.6%) sometimes doing so. Overall, 129 (58.1%) respondents tried to calm or support anxious patients. However, formal training in patient communication or holistic care was limited, as 183 (82.4%) respondents reported not attending any related workshop or training. The chi-square test indicated statistically significant differences in the distribution of answers for all five practice-related items (p < 0.05) (Table 7). While Q2 (χ² = 1.7, p < 0.05) and Q3 (χ² = 4.1, p < 0.05) did not exhibit statistically significant differences, Q1 (χ² = 6.2, p < 0.05), Q4 (χ² = 11, p < 0.05), and Q5 (χ² = 15, p < 0.05) demonstrated a statistically significant association between profession and practice of holistic care (Table 8).

**Table 7:** Practice of holistic healthcare. Data mentioned in the table represent the number of healthcare providers (n) and the percentage (%). *: χ² value represents the chi-square test statistic; **: p-value <0.05 is considered statistically significant.

Practice questions	Always	Often	Sometimes	Rarely	Statistical test	P-value**
Q1. How often do you ask patients about their worries or emotional problems?	140 (63.1%)	42 (18.9%)	40 (18.0%)	0 (0) %	χ²* = 87.35	<0.001
Q2. Do you involve the patient’s family while explaining the treatment or care?	152 (68.5%)	47 (21.2%)	22 (9.9%)	1 (0.5%)	χ²* = 204.47	<0.001
Q3. Do you consider the patient's language, culture, or beliefs while caring for them?	92 (41.4%)	40 (18.0%)	88 (39.6%)	2 (0.9%)	χ²* = 89.50	<0.001
Q4. When a patient is scared or anxious, do you try to calm or support them?	129 (58.1%)	34 (15.3%)	59 (26.6%)	0 (0)%	χ²* = 65.95	<0.001
Q5. Have you attended any training or workshop on patient communication or holistic care?	Yes 29 (13.1%)	No 183 (82.4%)	Not sure 10 (4.5%)	–	χ²* = 221.54	<0.001

**Table 8:** Profession-wise practice of holistic healthcare. Data mentioned in the table represent the number of doctors and nurses (n) and the percentage (%). *: χ² value represents the chi-square test statistic; **: p-value <0.05 is considered statistically significant.

Question	Profession	Always	Often	Sometimes	Rarely	Statistical test	P-value**
Q1. How often do you ask patients about their worries or emotional problems?	Doctor (n = 128)	82 (64.1%)	30 (23.4%)	16 (12.5%)	0 (0%)	χ²* = 6.2	0.044
	Nurse (n = 94)	58 (61.7%)	12 (12.8%)	24 (25.5%)	0 (0%)		
Q2. Do you involve the patient’s family while explaining the treatment or care?	Doctor (n = 128)	89 (69.5%)	29 (22.7%)	10 (7.8%)	0 (0%)	χ²* = 1.7	0.63
	Nurse (n = 94)	63 (67.0%)	18 (19.1%)	12 (12.8%)	1 (1.1%)		
Q3. Do you consider the patient’s language, culture, or beliefs while caring for them?	Doctor (n = 128)	58 (45.3%)	25 (19.5%)	45 (35.2%)	0 (0%)	χ²* = 4.1	0.25
	Nurse (n = 94)	34 (36.2%)	15 (16.0%)	43 (45.7%)	2 (2.1%)		
Q4. When a patient is scared or anxious, do you try to calm or support them?	Doctor (n = 128)	87 (68.0%)	20 (15.6%)	21 (16.4%)	0 (0%)	χ²* = 11	0.003
	Nurse (n = 94)	42 (44.7%)	14 (14.9%)	38 (40.4%)	0 (0%)		
Question	Profession	Yes, n (%)	No, n (%)	Not sure, n (%)	-	Statistical test	P-value**
Q5. Have you attended any training/workshop on patient communication or holistic care?	Doctor (n = 128)	1 (0.8%)	78 (60.9%)	6 (4.7%)	-	χ²* = 15	0.0007
	Nurse (n = 94)	19 (20.2%)	105 (81.4%)	4 (4.3%)	-		

## Discussion

A holistic healthcare approach to wellness covers the mental, emotional, social, spiritual, and physical aspects of health all at once. In keeping with the trend of examining the motivation behind wellness, holistic approaches to wellness should be taken into account. A holistic approach to wellness is an all-encompassing, integrated strategy that takes into account several facets of a person’s well-being and ignores the notions of wellness and health that could be impeding the development of practical solutions [8]. This questionnaire-based prospective survey was conducted to analyze the knowledge, attitudes, and practices of healthcare professionals regarding the holistic healthcare approach at a tertiary care teaching hospital.

In our study, among 222 participants, 129 (58.1%) were male, and 93 (49.1%) were female, which is in contrast to the study reported by Tsai et al. [9], where 257 (71.4%) females and 103 (28.6%) males were included. In our study, most participants were aged between 20 and 29 years (113, 50.9%), whereas in the study by Tsai et al. [9], the majority of the participants belonged to the 41-50-year age group (123, 34.2%). Among the participants, the majority had 1-5 years of experience (110, 49.5%), whereas in the study by Tsai et al. [9], 168 (46.7%) had more than 10 years of experience. In our study, the majority of participants were doctors (128, 57.7%), while 94 (42.3%) were nurses, whereas in the study reported by Awny et al. [10], 52 (23.6). were resident doctors.

In our study, the mean knowledge score among healthcare providers was 98.9%, indicating a very high level of awareness regarding holistic patient care. This finding compares favorably with previous studies conducted by Khasoha et al. [11] (50%). The substantially higher knowledge observed in our study may reflect improved access to continuing education, greater emphasis on professional development, and enhanced institutional support for holistic care practices. In the present study, no statistically significant association was observed between gender and knowledge levels regarding holistic care. Similarly, professions were also not significantly associated with knowledge scores among healthcare professionals. These results align with the research conducted by Albaqawi et al. [12], who also reported no significant differences in knowledge levels based on gender and profession among healthcare providers.

The findings of our study indicate a predominantly positive attitude toward holistic patient care among participants. A substantial proportion of respondents agreed that emotional support is as important as medications, with 45.5% strongly agreeing and 52.7% agreeing. These results are broadly comparable to the study by Khasoha et al. [11], in which 20% strongly agreed, and 75% agreed, underscoring the recognized importance of emotional support in patient management. Furthermore, most participants acknowledged the benefits of holistic care, with 51.4% agreeing that it contributes to faster recovery and improved well-being. This level of agreement is higher than that reported by Khasoha et al. [11], where 37.5% of respondents agreed. In our study, 48.6% of respondents agreed that work pressure and time constraints make the provision of holistic care challenging. In comparison, in the study by Khasoha et al. [11], 75% strongly agreed. In this study, the responses to all five attitude statements were statistically significant (χ² test, p < 0.05), indicating that participants predominantly agreed with statements supporting the importance of holistic care.

In our study, most participants reported positive patient-centered practices. The majority stated that they always ask patients about emotional concerns (63.1%), which is higher than the findings reported by Ambushe et al. [13] (53.2%). Similarly, 58.1% of respondents reported that they calm or support anxious patients, comparable to the 54% reported by Ambushe et al. [13]. These findings reflect attention to the psychological dimension of holistic care. Consideration of patients’ language, culture, and beliefs was also commonly reported, with 41.4% always and 39.6% sometimes addressing these aspects. This aligns with the findings of Ambushe et al. [13], who reported 54% in this domain, highlighting the importance of the sociocultural dimension of holistic care. However, despite these positive practices, formal training in patient communication or holistic care was limited.

In the present study, all five practice-related questions showed statistically significant differences (p < 0.05), indicating that healthcare professionals demonstrated consistently strong holistic care practices across multiple domains. These included asking patients about their emotional concerns, involving family members in treatment discussions, considering patients’ cultural and linguistic backgrounds, providing emotional reassurance to anxious patients, and participation in training related to patient communication or holistic care. In contrast, a study conducted by Argus-Calvo et al. [14] reported that only the psychological domain showed a statistically significant difference, while other domains did not reach statistical significance.

Strengths

One of the major strengths of our study is that it explores an area where limited data are available, particularly regarding the knowledge, attitudes, and practices of healthcare providers toward holistic healthcare. Despite the increasing emphasis on patient-centered care, there remains a scarcity of research evaluating how healthcare professionals integrate holistic principles into routine clinical practice.

Limitations

Our research provides important information about healthcare providers’ knowledge, attitudes, and practices regarding holistic healthcare. However, it has certain limitations. The results may not be as applicable to other healthcare settings, such as private clinics and rural hospitals, where exposure to and implementation of holistic healthcare practices may differ significantly, as the research was conducted at a single tertiary care teaching hospital. A potential ceiling effect was observed, as many participants correctly answered the knowledge items, suggesting that the questions may have been too basic to differentiate varying levels of knowledge. Additionally, the relatively small sample size may not adequately represent the broader population.

## Conclusions

The present study shows that healthcare providers have good knowledge of holistic healthcare, with most participants correctly identifying its meaning and key aspects. Overall attitudes were positive, as many agreed that emotional support is as important as medication and that holistic care improves recovery and well-being. In practice, a majority reported regularly addressing patients’ emotional concerns, involving family members, supporting anxious patients, and considering language, culture, and beliefs. However, formal training in holistic care and patient communication was limited, and work pressure was identified as a barrier. Therefore, conducting regular continuing medical education sessions, training programs, workshops, and skill-based sessions on holistic healthcare is recommended to strengthen practical competencies and ensure consistent, high-quality holistic care delivery.
